# Combined morphology and distribution features for risk stratification in pathology-enriched BI-RADS 4A/4B microcalcifications

**DOI:** 10.1186/s12885-026-16577-z

**Published:** 2026-08-13

**Authors:** Shiyi Zhang, Shiwen Dong, Yongtian Chen, Yonglong Yu, Wen Yang, Wenyan Guo, Qiuhong Lan, Guojie Huang, Yangyu Chen, Wenxin Chen

**Affiliations:** 1 Department of Breast Surgery, Sanming First Hospital Affiliated to Fujian Medical University, 29 Liedong Road, Sanming, 365001 Fujian China; 2 Fujian Medical University, Fuzhou, 350001 Fujian China; 3 Department of Medical Imaging, Sanming First Hospital Affiliated to Fujian Medical University, 29 Liedong Road, Sanming, 365001 Fujian China; 4 Center for Computing & Data Sciences, Boston University, 665 Commonwealth Ave, Boston, MA 02215 USA

**Keywords:** Breast cancer, Digital mammography, Microcalcifications, BI-RADS, Morphology, Distribution, Diagnostic accuracy

## Abstract

**Background:**

Breast microcalcifications are a common indication for biopsy in breast cancer screening and often represent the earliest mammographic manifestation of malignancy. However, substantial heterogeneity within BI-RADS 4 A lesions presents challenges for clinical interpretation. This study aimed to evaluate the association of microcalcification morphology and distribution with pathological outcomes and to explore their potential role in characterizing heterogeneity within BI-RADS 4 A lesions.

**Methods:**

A total of 327 patients with 356 pathologically confirmed microcalcification-positive breast lesions detected on digital mammography were retrospectively analyzed on a lesion-based basis. Calcification morphology and distribution were classified according to the BI-RADS 5th Edition. Associations with pathological outcomes were assessed using chi-square or Fisher’s exact tests. Multivariable logistic regression was performed to identify independent factors associated with malignancy. Receiver operating characteristic (ROC) curve analysis was used to evaluate diagnostic performance at BI-RADS thresholds ≥4 A and ≥4B.

**Results:**

Among the 327 patients, 205 (62.7%) had malignant disease and 122 (37.3%) had benign disease. Fine pleomorphic (80.2%), amorphous (66.2%), and fine linear or branching (100%) morphologies, as well as clustered (71.2%), regional (96.6%), and linear (100%) distribution patterns, were significantly associated with malignancy (*P* < 0.001). Multivariable analysis demonstrated that morphology and distribution remained independently associated with malignancy.

Among lesions initially categorized as BI-RADS 4 A in institutional practice (*n* = 58), substantial heterogeneity was observed. Lesions with high-risk morphology and distribution patterns were more frequently malignant, whereas punctate calcifications with scattered distribution showed a lower malignancy proportion. In this pathology-confirmed cohort, BI-RADS ≥4B showed higher specificity than BI-RADS ≥4 A, although this category-level finding should be interpreted descriptively.

**Conclusions:**

In this retrospective cohort of biopsied BI-RADS 4 A/4B microcalcification-positive lesions, combined morphology and distribution features provided supplementary information for imaging–pathology correlation and helped illustrate risk heterogeneity within institutionally assigned BI-RADS 4 A lesions. These findings should be interpreted as confirmatory observations within a selected biopsy cohort rather than population-level malignancy estimates, and they are not intended to replace established BI-RADS-based management principles.

**Clinical implications:**

These findings should be interpreted with caution due to the pathology-enriched design and should not be considered population-level risk estimates. They may provide supplementary information for understanding lesion heterogeneity but are not intended to replace established BI-RADS-based clinical decision-making.

## Highlights

This retrospective study analyzed 356 pathologically confirmed microcalcification-positive breast lesions detected on digital mammography.

Fine pleomorphic and fine linear or branching microcalcification morphologies showed the strongest association with malignancy.

Clustered, regional, and linear distribution patterns were significantly correlated with higher malignancy rates.

BI-RADS 4 A lesions exhibited substantial internal heterogeneity in this pathology-enriched cohort.

Morphology and distribution together may provide supplementary information for interpreting lesions initially categorized as BI-RADS 4 A.

## Introduction

Breast cancer remains the most frequently diagnosed malignancy among women worldwide and a leading cause of cancer-related death. According to the most recent GLOBOCAN 2022 estimates, breast cancer continues to account for a substantial proportion of the global cancer burden among women [1]. Early detection and timely intervention are key to improving survival rates, with five-year survival exceeding 95% in stage I breast cancer compared to less than 30% in advanced stages [2]. Therefore, enhancing early diagnostic accuracy has become a central focus of breast imaging research.

Digital mammography (DM) is the cornerstone of population-level breast cancer screening. Large cohort analyses and guideline statements have consistently demonstrated that regular mammographic screening can reduce breast cancer mortality [3, 4]. Nonetheless, optimizing the balance between sensitivity and specificity—particularly reducing unnecessary biopsies without compromising early cancer detection—remains a critical challenge in daily practice.

The Breast Imaging Reporting and Data System (BI-RADS) was introduced to standardize terminology, improve communication, and refine clinical decision-making. Despite these advantages, considerable inter- and intra-observer variability persists in BI-RADS feature descriptors and final assessment categories, particularly within the low-suspicion BI-RADS 4 A category [5]. The 5th edition BI-RADS Atlas updated imaging descriptors for calcification morphology and distribution, but real-world risk calibration still benefits from additional evidence-based refinement [6].

Among mammographic findings, microcalcifications are especially relevant because they often represent the earliest imaging correlates of ductal carcinoma in situ (DCIS) or early invasive disease. Numerous studies have demonstrated that calcification morphology and distribution serve as robust predictors of malignancy. Fine pleomorphic and fine linear/branching morphologies exhibit the highest positive predictive value (PPV) for cancer, whereas coarse, round, or rim calcifications typically indicate benign conditions [7, 8]. Likewise, linear, segmental, or clustered distributions are more frequently associated with malignant ductal spread, whereas scattered distributions are generally considered more often benign [9].

Despite these insights, the BI-RADS 4 A category—intended to represent low suspicion for malignancy (≈ 2–10%)—remains highly heterogeneous in clinical practice. Several recent studies have proposed refined scoring systems based on BI-RADS 5th edition descriptors [10], while others have suggested that certain morphologies such as amorphous or punctate calcifications may be managed with short-interval follow-up in the presence of benign-oriented distributions [11]. Translating these approaches into routine clinical management requires validation using pathology-confirmed, real-world datasets. However, it is important to note that findings derived from pathology-confirmed cohorts may not be directly comparable to those from large screening-based populations, where the full spectrum of low-risk and non-biopsied lesions is represented.

Against this backdrop, the present study retrospectively analyzed pathologically verified microcalcification-positive lesions from a tertiary medical center. The primary purpose of this study was not to redefine the established predictive value of BI-RADS calcification descriptors, but rather to evaluate how morphology and distribution patterns manifest within a pathology-enriched real-world cohort of biopsied BI-RADS 4 A/4B lesions.

Specifically, we aimed to:

1. characterize imaging–pathology associations of calcification morphology and distribution in biopsied BI-RADS 4 A/4B lesions;
2. explore whether combined morphology and distribution patterns may help illustrate heterogeneity among lesions initially categorized as BI-RADS 4 A in institutional practice; and.
3. descriptively evaluate diagnostic performance across BI-RADS thresholds within this retrospective pathology-confirmed cohort.

## Materials and methods

### Study design and ethical approval

This retrospective study was conducted in the Department of Breast Surgery, Sanming First Hospital, Fujian Medical University. The study protocol was approved by the Institutional Ethics Review Board (Approval No. Ming Yi Lun [2024] No. 110). The requirement for informed consent was waived due to the retrospective design of the study and the anonymized nature of the data, in accordance with the Declaration of Helsinki [12].

### Patient selection

**Fig. 1:**
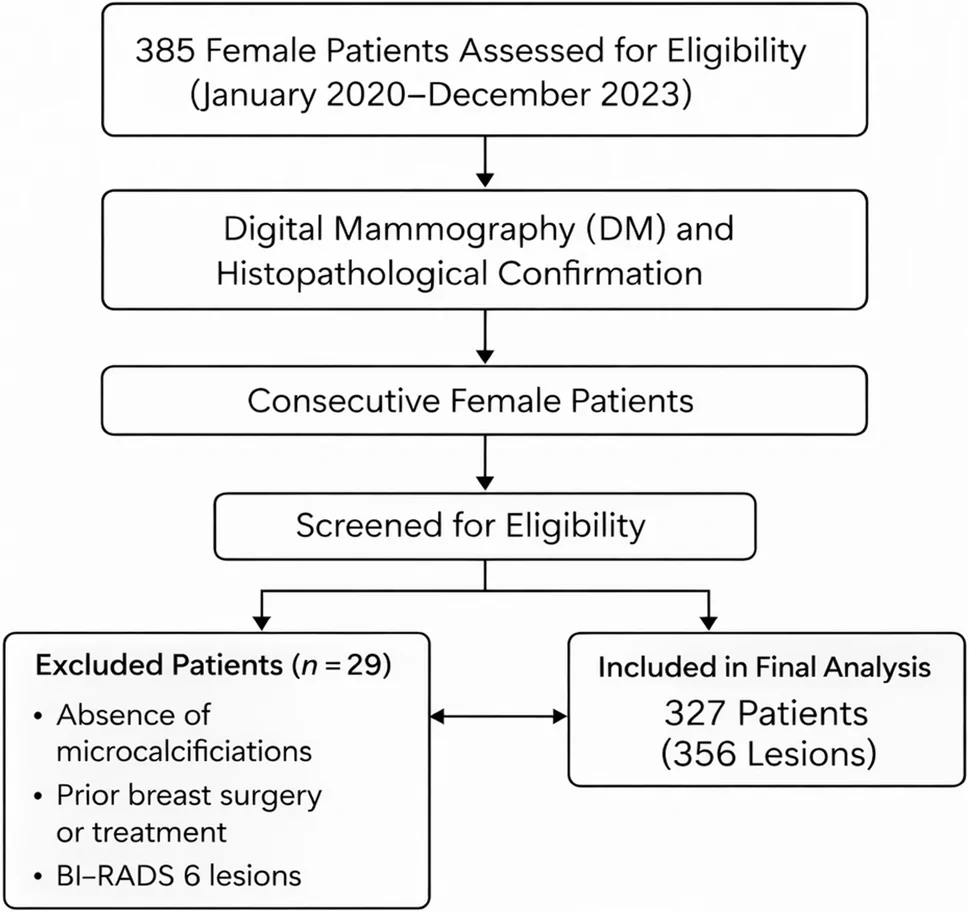
Flowchart of patient selection and study inclusion

This was a retrospective single-center study. Consecutive eligible female patients who underwent digital mammography (DM) followed by histopathological confirmation between January 2020 and December 2023 were retrospectively identified from the institutional database. A total of 385 female patients were initially assessed, of whom 29 were excluded according to the predefined criteria. The final analysis included 327 patients with 356 microcalcification-positive lesions, as some patients presented with bilateral or multifocal lesions (Fig. 1).

### Inclusion criteria

1. presence of microcalcifications identified on full-field digital mammography (FFDM);
2. availability of complete pathological results (core needle biopsy or surgical excision);
3. imaging performed according to BI-RADS 5th edition (2013) classification.

### Exclusion criteria

1. absence of visible calcifications on mammography;
2. prior breast surgery, radiotherapy, or chemotherapy;
3. BI-RADS 6 category (pathology-proven malignancy prior to imaging).

### Imaging acquisition and analysis

Digital mammograms were acquired using a Hologic Selenia Dimensions system (Hologic Inc.) with automatic exposure control. Standard craniocaudal (CC) and mediolateral oblique (MLO) views were obtained for both breasts.

Two senior radiologists (each with more than 10 years of experience in breast imaging) independently reviewed all images, and a consensus on the final classification was reached. Discrepancies were resolved by a third senior radiologist. Radiologists were blinded to the final pathological diagnosis during image interpretation.

Microcalcification morphology and distribution patterns were classified according to the ACR BI-RADS 5th Edition descriptors. Morphology was categorized as fine pleomorphic, amorphous, fine linear/branching, coarse heterogeneous, punctate, ring-like (rim), or coarse. Distribution patterns were categorized as clustered, regional, linear, segmental, or scattered based on retrospective image review.

Each case was assigned a BI-RADS category (3–5) based on the overall imaging impression. BI-RADS 4 subclassification (4 A, 4B, and 4 C) was determined using integrated radiologic assessment in routine clinical practice and retrospective review, taking into account morphology, distribution, lesion extent, and overall imaging appearance.

Table 1 summarizes the general interpretive tendencies used for BI-RADS 4 subclassification in our institutional practice. In general, BI-RADS 4 A was assigned to lesions with relatively limited suspicious features on integrated assessment, whereas BI-RADS 4B and 4 C reflected progressively greater levels of radiologic concern based on the combined evaluation of morphology, distribution, lesion extent, and overall imaging context.

Highly suspicious findings, such as fine linear/branching morphology or suspicious ductal-oriented distribution, more often contributed to assignment into higher BI-RADS 4 subcategories. However, these subclassifications were based on integrated real-world radiologic interpretation rather than strict rule-based criteria and may reflect some degree of institutional and inter-reader variability.

Although the clinical motivation of this study was largely related to heterogeneity within BI-RADS 4 A lesions, BI-RADS 4B lesions were also included to provide an adjacent intermediate-suspicion comparison group within the biopsy pathway. The BI-RADS 4 A subgroup was analyzed separately to address the specific question of within-category heterogeneity.

In routine clinical practice, biopsy recommendation was based on integrated radiologic assessment rather than calcification morphology or distribution alone. For lesions with scattered calcifications, biopsy targeted the most suspicious or representative calcification focus identified during radiologic assessment. Lesions rated BI-RADS 4 A, 4B, and 4 C were considered suspicious and included in further analyses.

For multivariable analysis, morphology and distribution were further grouped into clinically meaningful risk-based categories to improve model stability and reduce sparse-category bias. Morphology was categorized as low-risk (punctate, coarse, and rim), intermediate-risk (amorphous and coarse heterogeneous), and high-risk (fine pleomorphic and fine linear/branching). Distribution patterns were categorized as low-risk (scattered), intermediate-risk (clustered), and high-risk (linear and segmental), based on BI-RADS descriptors and their known association with malignancy.

Prior mammographic examinations and temporal stability were not consistently available and were therefore not included as standardized variables.

**Table 1:** General interpretive principles for BI-RADS 4 subclassification in our institutional practice

BI-RADS subcategory	General interpretive tendencies in institutional practice	Typical level of concern
4 A	Relatively limited suspicious features on integrated assessment; mildly suspicious morphology, limited extent, or relatively benign-oriented distribution without more concerning associated findings	Low suspicion
4 B	More evident suspicious morphology or distribution, greater lesion extent, or combined imaging findings suggesting intermediate concern for malignancy	Intermediate suspicion
4 C	Highly suspicious imaging features, including marked suspicious morphology or ductal-oriented distribution patterns, but without classic findings sufficient for BI-RADS 5	Moderate-to-high suspicion

### Pathological diagnosis

Histopathological confirmation was performed by two senior pathologists blinded to imaging findings. Diagnoses were classified as benign (e.g., fibroadenoma, sclerosing adenosis, fibrocystic change) or malignant (including ductal carcinoma in situ [DCIS], invasive ductal carcinoma [IDC], and invasive lobular carcinoma [ILC]).

For DCIS cases, nuclear grade (low/intermediate/high) and presence of comedo necrosis were recorded according to the WHO Classification of Tumours of the Breast (5th edition, 2019).

### Statistical analysis

All statistical analyses were performed using SPSS version 26.0 (IBM Corp., Armonk, NY, USA) and R version 4.3.1. Analyses were conducted on a lesion-based basis, with each microcalcification-positive lesion considered an independent analytical unit unless otherwise specified.

Categorical variables were summarized as counts and percentages. Comparisons between malignant and benign lesions were performed using the chi-square test or Fisher’s exact test, as appropriate. For contingency tables with small expected cell counts, Fisher’s exact test with Monte Carlo simulation was applied. Lesions with indeterminate or unclassifiable morphology or distribution were excluded from the corresponding subgroup analyses.

Diagnostic performance of BI-RADS thresholds (≥ 4 A and ≥4B) was evaluated using receiver operating characteristic (ROC) curve analysis. Sensitivity, specificity, positive predictive value (PPV), negative predictive value (NPV), and area under the curve (AUC) with 95% confidence intervals (CIs) were calculated.

To improve model stability and reduce sparse-category bias, morphology and distribution were grouped into clinically meaningful risk-based categories (low-, intermediate-, and high-risk). Multivariable logistic regression was performed to identify independent factors associated with malignancy, including age, morphology group, and distribution group. Odds ratios (ORs) with 95% CIs were reported.

A two-sided *P* value < 0.05 was considered statistically significant.

## Results

### Patient characteristics

A total of 327 female patients with breast microcalcifications confirmed by pathology were included in this study. The mean age was 48.6 ± 9.8 years (range: 27–78 years). The cohort was derived from routine clinical practice at a tertiary medical center and likely included both screening-detected and clinically referred patients; however, the indication for mammography was not uniformly available in the retrospective records. Among the 327 patients, 205 (62.7%) were diagnosed with malignant disease and 122 (37.3%) with benign disease. Patient-based classification was used for baseline description; all subsequent analyses were performed on a lesion-based basis (*n* = 356 lesions), unless otherwise specified.

The malignant group included 153 invasive ductal carcinomas (IDC, 74.6%), 34 ductal carcinoma in situ (DCIS, 16.6%), and 18 invasive lobular carcinomas (ILC, 8.8%).

Benign lesions consisted primarily of fibrocystic changes (38.5%), sclerosing adenosis (25.4%), and fibroadenoma (22.1%).

### Relationship between microcalcification morphology and pathological results

**Fig. 2:**
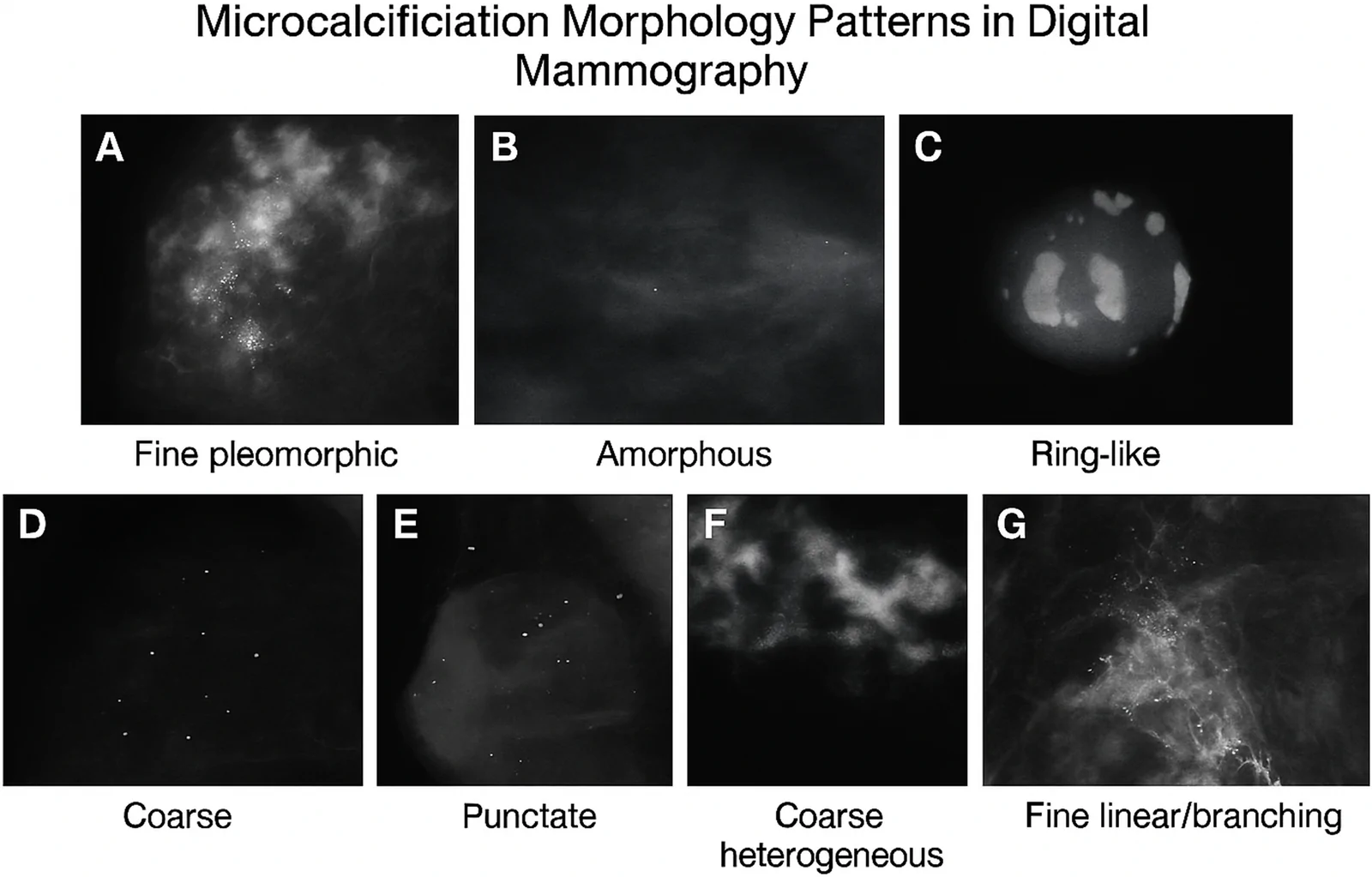
Representative microcalcification morphology patterns in digital mammography. **A** Fine pleomorphic calcifications, appearing as numerous small, irregular and variably sized high-density foci, strongly suggestive of malignancy. **B** Amorphous calcifications, showing faint, powdery and indistinct densities without a clearly defined shape, commonly associated with low-grade DCIS or benign proliferative changes. **C** Ring-like calcifications, characterized by curvilinear or circumferential calcified rims surrounding a radiolucent center, usually related to benign cystic or secretory processes. **D** Coarse calcifications, large, dense and relatively well-defined deposits typically resulting from degenerative or secretory benign changes. **E** Punctate calcifications, tiny, round, well-defined high-density foci most often associated with benign conditions. **F** Coarse heterogeneous calcifications, irregular, variably sized dense foci indicating an intermediate level of suspicion for malignancy. **G** Fine linear or fine branching calcifications, extremely thin, thread-like or branching high-density foci following ductal structures, highly predictive of high-grade DCIS

Five lesions with indeterminate or unclassifiable morphology were excluded from the morphology-based analysis; therefore, 351 lesions were included in the analysis presented in Table 2. Significant differences were observed in the distribution of microcalcification morphologies between malignant and benign lesions (Fisher’s exact test with Monte Carlo simulation, *P* < 0.001). Representative microcalcification morphologies are shown in Fig. 2.

**Fig. 3:**
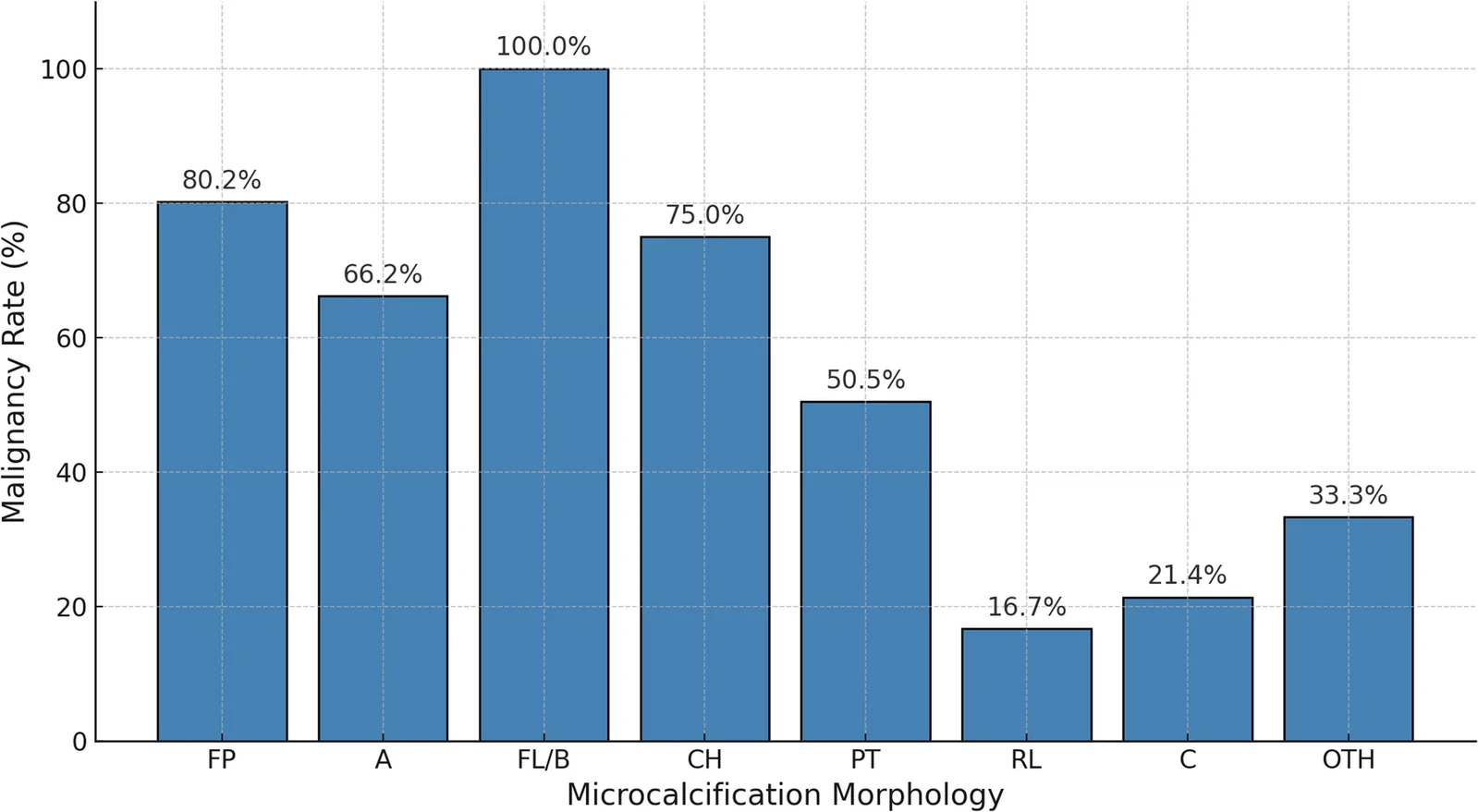
Relationship between microcalcification morphology and malignancy rate. Fine pleomorphic (FP), amorphous (A), and fine linear/branching (FL/B) calcifications showed the highest malignancy rates, whereas ring-like (RL) and coarse (C) types were predominantly benign. Abbreviations: FP, fine pleomorphic; A, amorphous; FL/B, fine linear or branching; CH, coarse heterogeneous; PT, punctate; RL, ring-like; C, coarse; OTH, others

Fine pleomorphic (80.2%), amorphous (66.2%), and fine linear/branching (100%) calcifications were predominantly associated with malignancy, whereas ring-like (16.7%) and coarse (21.4%) calcifications were mostly benign (Table 2; Fig. 3).

**Table 2:** Relationship between microcalcification morphology and pathological results (*n* = 351 lesions)

Morphology type	Malignant (*n*)	Benign (*n*)	Malignant rate (%)	Clinical implication
Fine pleomorphic	81	20	80.2	Strongly suggestive of malignancy
Amorphous	45	23	66.2	Suspicious tendency
Fine linear/branching	9	0	100	Strong malignant association
Coarse heterogeneous	9	3	75.0	Higher malignancy proportion
Punctate	52	51	50.5	Nonspecific; correlate with distribution
Ring-like	3	15	16.7	Benign tendency
Coarse	6	22	21.4	Benign tendency
Others	4	8	33.3	Indeterminate

Fisher’s exact test with Monte Carlo simulation, *P* < 0.001

### Relationship between microcalcification distribution and pathological results

Thirty lesions with indeterminate or unclassifiable distribution patterns were excluded from the distribution-based analysis; therefore, 326 lesions were included in the analysis presented in Table 3. Microcalcification distribution patterns were significantly associated with malignancy (Fisher’s exact test with Monte Carlo simulation, *P* < 0.001). Lesions with regional (96.6%), clustered (71.2%), segmental (88.9%), or linear (100%) distributions were more frequently associated with malignancy, whereas scattered distributions showed the lowest malignancy rate among the analyzed distribution categories (39.8%). (Table 3).

**Fig. 4:**
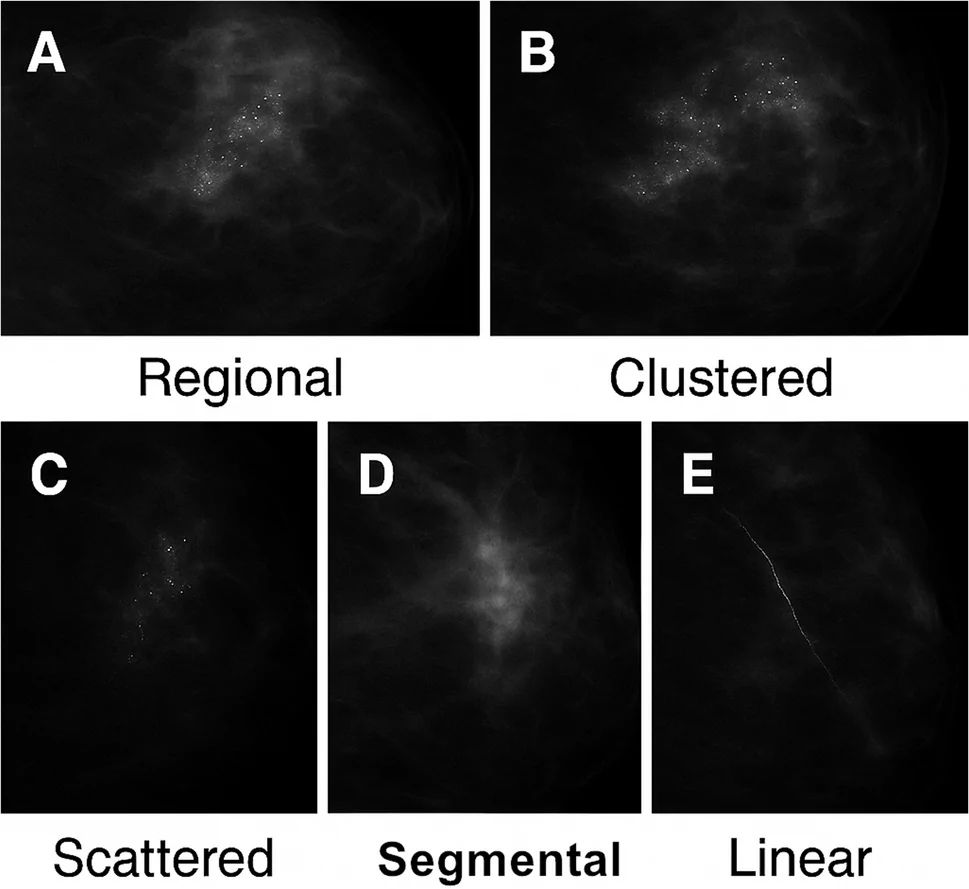
Distribution patterns of breast microcalcifications on digital mammography. Representative images demonstrate five distinct distribution types: (**A**) Regional, (**B**) Clustered, (**C**) Scattered, (**D**) Segmental, and (**E**) Linear.Each pattern illustrates the spatial arrangement of calcifications within the breast parenchyma, which aids in differentiating benign from malignant lesions in radiologic assessment

Representative mammographic appearances of these distribution types are shown in Fig. 4.

**Table 3:** Relationship between microcalcification distribution patterns and pathological results (*n* = 326 lesions)

Distribution pattern	Malignant (*n*)	Benign (*n*)	Malignant rate (%)
Regional	28	1	96.6
Clustered	114	46	71.2
Scattered	49	74	39.8
Segmental	8	1	88.9
Linear	6	0	100

Fisher’s exact test with Monte Carlo simulation, *P* < 0.001

### Subgroup analysis of BI-RADS 4 A lesions

Among 58 BI-RADS 4 A lesions, 17 (29.3%) were malignant and 41 (70.7%) were benign. The malignancy rate differed significantly across morphology–distribution combinations (Fisher’s exact test with Monte Carlo simulation, *P* < 0.01). Lesions with fine pleomorphic or amorphous morphology combined with clustered, regional, or linear distributions showed higher malignancy rates, whereas punctate or coarse/ring-like calcifications with scattered distribution were more often benign (Table 4).

**Table 4:** Microcalcification morphology–distribution combinations and pathology in BI-RADS 4 A lesions (*n* = 58)

Morphology	Distribution	Malignant	Benign	Malignant rate (%)	Observed tendency
Fine pleomorphic	Clustered/Regional/Linear	7	4	63.6	Higher malignancy proportion
Amorphous	Clustered/Linear	3	4	42.9	Intermediate-to-higher risk tendency
Coarse heterogeneous	Clustered	1	2	33.3	Indeterminate
Fine pleomorphic	Scattered	1	2	33.3	Indeterminate
Punctate	Clustered/Segmental	2	6	25.0	Lower malignancy proportion
Punctate	Scattered	3	20	13.0	More often benign
Coarse/Ring-like	Scattered	0	3	0	Benign tendency

P value calculated using Fisher’s exact test with Monte Carlo simulation (*P* < 0.01)

### Diagnostic performance of BI-RADS thresholds

ROC analysis was performed in 318 lesions with complete BI-RADS categorization and pathological reference data; lesions with missing or indeterminate BI-RADS assessments were excluded.

Using pathology as the reference standard, diagnostic performance was analyzed for BI-RADS ≥4 A and ≥4B thresholds (Table 5).

BI-RADS ≥4 A achieved high sensitivity (96.3%) but low specificity (48.0%), while BI-RADS ≥4B showed balanced sensitivity (89.0%) and higher specificity (81.1%).

**Fig. 5:**
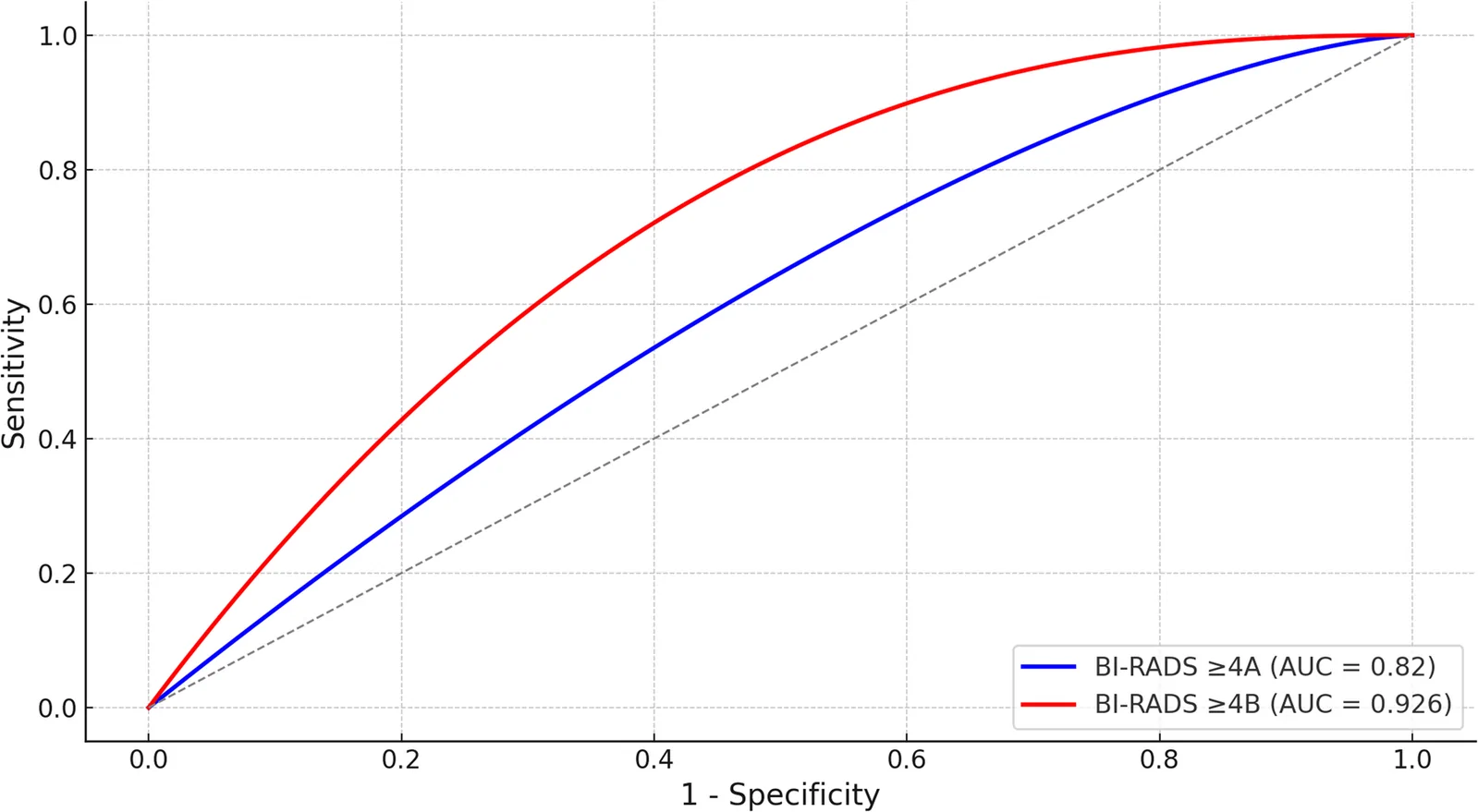
Receiver operating characteristic (ROC) curves for BI-RADS ≥4 A and ≥4B thresholds. The ROC curve for the BI-RADS ≥4B threshold showed a higher AUC (0.926) than that for the ≥4 A threshold (0.82)

The BI-RADS ≥4B threshold showed a higher AUC (0.926) than the ≥4 A threshold (0.820), suggesting a more favorable balance between sensitivity and specificity within this pathology-confirmed cohort (Fig. 5). A formal statistical comparison between AUCs was not performed due to the retrospective design and limited sample size, and the ROC results should be interpreted descriptively.

**Table 5:** Diagnostic performance of BI-RADS thresholds for malignancy classification (*n* = 318 lesions)

Threshold	TP	FP	FN	TN	Sensitivity	Specificity	PPV	NPV	AUC
≥ 4 A	184	66	7	61	96.3%	48.0%	73.6%	89.7%	0.820
≥ 4B	170	24	21	103	89.0%	81.1%	87.6%	83.1%	0.926

### Multivariable logistic regression analysis

To evaluate independent factors associated with malignancy, multivariable logistic regression analysis was performed after grouping morphology and distribution into clinically meaningful risk-based categories.

After adjustment for age and distribution, high-risk morphology remained independently associated with malignancy compared with low-risk morphology (OR = 2.156, 95% CI: 1.017–4.573, *P* = 0.045), whereas intermediate-risk morphology was not significantly associated with malignancy (OR = 1.151, 95% CI: 0.535–2.477, *P* = 0.719).

Similarly, both intermediate-risk distribution (OR = 2.992, 95% CI: 1.516–5.903, *P* = 0.002) and high-risk distribution (OR = 12.565, 95% CI: 1.506–104.844, *P* = 0.019) were independently associated with malignancy compared with low-risk distribution.

Age was also independently associated with malignancy (OR = 1.059, 95% CI: 1.027–1.091, *P* < 0.001).

Detailed results are presented in Table 6.

**Table 6:** Multivariable logistic regression analysis of factors associated with malignancy in microcalcification-positive breast lesions

Variable	OR	95% CI	*P* value
Age	1.059	1.027–1.091	< 0.001
Morphology: intermediate-risk vs. low-risk	1.151	0.535–2.477	0.719
Morphology: high-risk vs. low-risk	2.156	1.017–4.573	0.045
Distribution: intermediate-risk vs. low-risk	2.992	1.516–5.903	0.002
Distribution: high-risk vs. low-risk	12.565	1.506–104.844	0.019

Low-risk categories were used as the reference group. The model included age, morphology group, and distribution group

Abbreviations: *OR* odds ratio, *CI* confidence interval

## Discussion

In this retrospective real-world cohort of biopsied microcalcification-positive breast lesions, our findings were generally consistent with established BI-RADS principles and prior radiologic–pathologic studies. Importantly, multivariable logistic regression analysis demonstrated that both microcalcification morphology and distribution remained independently associated with malignancy after adjustment for age.

The recently updated BI-RADS 6th Edition (2025) further underscores the importance of standardized interpretation of calcification morphology and distribution. Although the present study was conducted using BI-RADS 5th Edition descriptors, the updated framework provides important context for interpretation of our findings. In particular, the revised definition of amorphous calcifications, the refined distinction between regional and segmental distribution patterns, updated positive predictive value expectations for BI-RADS 4 subclassification, and increased emphasis on temporal stability may help explain some of the variability observed in this retrospective cohort. The observed associations between morphology, distribution, and malignancy in our study are broadly consistent with the conceptual framework emphasized in the updated BI-RADS Atlas.

Accordingly, the unusually high malignancy rates observed in some categories, particularly regional distribution and institutionally assigned BI-RADS 4 A lesions, should not be interpreted as representative of screening populations. These findings may partially reflect the selected biopsy cohort design, retrospective interpretive overlap between regional and segmental patterns, and real-world inter-reader variability in BI-RADS assessment. Because the study population consisted exclusively of biopsied lesions with pathological confirmation, the observed malignancy rates represent risk estimates within a biopsy-enriched cohort and are not directly comparable to those reported in screening-based populations. Future prospective studies applying updated BI-RADS descriptors may provide more reliable risk calibration.

These findings support the independent contribution of morphology and distribution in imaging–pathology correlation within this cohort. Large screening-based studies have demonstrated that the predictive value of microcalcification patterns should be evaluated within unbiased populations. In contrast, the present study reflects imaging–pathology associations within a selected biopsy cohort rather than population-level malignancy risk estimates.

It is important to distinguish the interpretation of individual imaging descriptors from the performance of BI-RADS assessment categories. In the present study, morphology and distribution were evaluated as independent imaging features associated with pathological outcomes, whereas BI-RADS categories represent composite clinical assessments integrating multiple imaging findings, lesion extent, and contextual factors. Therefore, associations observed for specific calcification descriptors should not be interpreted as direct measures of BI-RADS category performance.

The main contribution of the present study lies not in redefining established BI-RADS principles, but in illustrating their manifestation in a real-world institutional cohort and highlighting the heterogeneity observed among lesions initially categorized as BI-RADS 4 A.

In the BI-RADS 4 A subgroup, combined consideration of morphological and distributional characteristics helped illustrate differences in malignancy likelihood among lesions initially assigned to the same category in institutional practice. ROC curve analysis further showed that institutionally assigned BI-RADS ≥4B categories provided a more favorable balance between sensitivity and specificity than BI-RADS ≥4 A within this selected pathology-confirmed cohort. These findings reflect category performance within the present cohort and should be interpreted separately from the independent imaging–pathology associations observed for morphology and distribution descriptors.

Collectively, these findings provide supplementary real-world evidence for interpretation of heterogeneous microcalcification-positive lesions encountered in institutional practice; however, prospective multicenter validation remains necessary before broader clinical application.

### Diagnostic significance of microcalcification morphology

The current study confirmed that fine pleomorphic, fine linear/branching, and amorphous calcifications are highly predictive of malignancy, consistent with prior literature. Bent et al. reported that fine linear/branching morphology had the highest positive predictive value (PPV) among BI-RADS descriptors on full-field digital mammography [7]. Similarly, Youk et al. developed a quantitative scoring system in which fine pleomorphic and fine linear/branching morphologies achieved the highest malignancy scores [10].Our findings align with these results, showing malignancy rates of 80.2% and 100% for fine pleomorphic and fine linear/branching calcifications, respectively.

In contrast, coarse (e.g., popcorn-like) and rim calcifications have been traditionally considered among the ‘typically benign’ types in mammography, frequently associated with degenerative or secretory breast conditions such as involuting fibroadenomas or fat-necrosis. However, given the recognized overlap and diagnostic uncertainty, benign appearance does not guarantee absence of neoplasia, and clinical judgement should be maintained, especially when other risk factors are present.

Recent studies have also emphasized the variable malignancy risk of amorphous calcifications depending on their context. Shen et al. constructed a nomogram model demonstrating that grouped amorphous calcifications, larger extent, and older age significantly increase malignancy likelihood [13]. This supports our finding that amorphous morphology, when combined with suspicious distribution, warrants closer evaluation.

These findings were further supported by multivariable analysis, in which high-risk morphology remained independently associated with malignancy after adjustment.

### Diagnostic significance of distribution pattern

Analysis of distribution patterns revealed that clustered, regional, and linear calcifications were strongly associated with malignancy, whereas scattered patterns showed a lower malignancy proportion than other distribution categories. Lai et al. observed that linear or segmental calcifications often correspond to intraductal spread of ductal carcinoma in situ (DCIS) [8], a finding generally consistent with our observations. These findings support the recognized association between ductal-oriented calcification patterns and malignant ductal spread.

Another notable finding was the unusually high malignancy rate observed in lesions classified as having regional distribution. This result should be interpreted with substantial caution, as it is considerably higher than that reported in most screening-based studies. Several factors may have contributed to this discrepancy. First, the present cohort was derived from a selected biopsy population, which likely inflated malignancy rates across all distribution categories. Second, distinction between regional and segmental distribution patterns may be challenging in retrospective image review, particularly in extensive or heterogeneous calcification clusters, potentially contributing to interpretive overlap and classification variability. In addition, institutional referral patterns and real-world inter-reader variability may also have influenced distribution categorization and biopsy selection.

Accordingly, the malignancy rate observed for regional distribution in this study should not be interpreted as representative of general screening populations or as evidence that regional distribution alone carries uniformly high malignant potential.

Similarly, distribution patterns remained independently associated with malignancy in multivariable analysis, consistent with their known relationship with malignant ductal spread.

### BI-RADS 4 A lesions and heterogeneity in real-world practice

A notable finding of this study was the heterogeneity observed among lesions initially categorized as BI-RADS 4 A, a subgroup traditionally associated with a low probability of malignancy (2–10%) [14]. In our cohort, the malignancy rate of BI-RADS 4 A lesions was 29.3%, which substantially exceeded the expected range reported in screening-based populations and should therefore be interpreted with considerable caution.

Several factors may have contributed to this elevated malignancy rate. First, the study cohort consisted exclusively of biopsied lesions with pathological confirmation, resulting in substantial selection bias and enrichment for suspicious findings. As a tertiary referral center, our institution may also receive a higher proportion of clinically concerning or radiologically ambiguous lesions, further increasing the apparent malignancy rate within BI-RADS 4 A cases.

In addition, BI-RADS subclassification in this retrospective real-world cohort was based on integrated radiologic interpretation in routine clinical practice rather than strict rule-based criteria. Accordingly, some degree of inter-reader variability and institutional interpretive heterogeneity likely contributed to overlap among BI-RADS 4 subcategories. Certain morphology–distribution combinations observed within the BI-RADS 4 A subgroup, including lesions with relatively suspicious imaging features, may therefore reflect real-world variability in radiologic assessment and biopsy decision-making.

Within the BI-RADS 4 A subgroup, lesions showing fine pleomorphic or amorphous morphology together with clustered, regional, or linear distribution patterns were more frequently malignant, whereas punctate or coarse/ring-like calcifications with scattered distribution were more often benign. Importantly, multivariable analysis further demonstrated that morphology and distribution remained independently associated with malignancy, suggesting that differences in these imaging features may partially contribute to heterogeneity within institutionally assigned BI-RADS 4 A lesions.

Rather than redefining the expected performance of BI-RADS 4 A, these findings primarily illustrate the variability and heterogeneity encountered in real-world institutional practice. Accordingly, the malignancy rate observed in this study should not be interpreted as representative of BI-RADS 4 A performance in general screening populations. Prospective multicenter studies applying standardized contemporary BI-RADS criteria are warranted for further validation. It should also be noted that the BI-RADS 4 A subgroup sample size was relatively small, and institutional variability in BI-RADS 4 A assignment may have influenced subgroup classification.

### Diagnostic threshold analysis

Receiver operating characteristic (ROC) analysis showed that, within this selected pathology-confirmed cohort, institutionally assigned BI-RADS ≥4B categories had higher specificity (81.1% vs. 48.0%) while maintaining relatively high sensitivity (89.0% vs. 96.3%) compared with BI-RADS ≥4 A. The ≥4B threshold showed a more favorable balance between sensitivity and specificity in this cohort. However, these results should be interpreted descriptively as category-level performance within a biopsy-enriched dataset, rather than as validation of BI-RADS threshold performance in broader screening populations. Whether such threshold differences could reduce unnecessary biopsies without affecting early cancer detection requires prospective validation in unselected or screening-based cohorts.

### Clinical implications

From a clinical perspective, the present findings should be interpreted as supplementary evidence from a pathology-enriched retrospective cohort rather than as a replacement for established BI-RADS principles. The observed morphology–distribution patterns may help illustrate the heterogeneity of microcalcification-positive lesions encountered in routine practice, particularly among lesions initially categorized as BI-RADS 4 A in our institutional setting. In this context, improving interpretation of heterogeneous calcification-positive lesions may be relevant to ongoing efforts to balance early cancer detection with the avoidance of overtreatment in breast screening practice [15]. These findings may support more cautious interpretation in selected cases, but should not be used in isolation to determine biopsy or follow-up strategies. In addition, these observations may provide hypothesis-generating information for future studies integrating mammographic imaging features into artificial intelligence– or radiomics-assisted diagnostic models [16, 17]. Further prospective multicenter validation is needed before broader clinical application.

### Limitations and future perspectives

Several limitations should be acknowledged. First, this was a single-center retrospective study, which may limit external generalizability. Second, the study cohort consisted exclusively of biopsied lesions with histopathological confirmation, resulting in substantial selection bias and enrichment for suspicious findings. Consequently, the malignancy rates observed in this study, including those for BI-RADS 4 A lesions and regional distribution patterns, should not be interpreted as representative of screening populations or as population-level estimates of malignancy risk.

Third, non-biopsied BI-RADS 2–3 lesions and lesions managed with imaging follow-up were not systematically included, limiting evaluation of the full spectrum of mammographically detected calcifications and preventing direct comparison with routine screening cohorts. In addition, because the indication for mammography was not uniformly available in the retrospective records, differences between screening-detected and clinically referred lesions could not be formally assessed and may also have influenced the observed malignancy rates.

Fourth, prior mammograms and temporal stability, which are important considerations in contemporary BI-RADS assessment, were not consistently available and therefore could not be incorporated into the analysis. Interobserver agreement for morphology and distribution assessment was also not formally evaluated, and retrospective image review may have introduced some degree of interpretive variability, particularly in distinguishing regional from segmental distribution patterns.

Finally, because some patients contributed more than one lesion, within-patient clustering may have influenced variance estimates. In addition, the relatively limited sample size in certain morphology and distribution subgroups may have contributed to statistical instability in some subgroup analyses.

In addition, subgroup analyses, particularly within BI-RADS 4 A lesions, were limited by relatively small sample sizes, and institutional variability in BI-RADS assignment may have influenced classification.

Future prospective multicenter studies applying contemporary BI-RADS descriptors and including both biopsied and non-biopsied lesions with longitudinal follow-up are warranted to further validate these findings.

## Conclusion

In this retrospective real-world cohort of biopsied microcalcification-positive breast lesions, calcification morphology and distribution patterns were associated with pathological outcomes and remained independently associated with malignancy in multivariable analysis. Fine pleomorphic, amorphous, and fine linear or branching morphologies, together with clustered, regional, or linear distribution patterns, were more frequently observed in malignant lesions.

Importantly, substantial heterogeneity was observed among lesions initially categorized as BI-RADS 4 A. Differences in morphology and distribution may partially contribute to this heterogeneity and may help illustrate variability encountered in real-world institutional practice.

Overall, these findings provide supplementary evidence for imaging–pathology correlation within a selected biopsy cohort. The associations observed for individual morphology and distribution descriptors should be interpreted separately from the performance of BI-RADS assessment categories. However, the observed malignancy rates, particularly those associated with regional distribution patterns and institutionally assigned BI-RADS 4 A lesions, should not be interpreted as representative of screening populations or as population-level risk estimates.

Rather than redefining established BI-RADS management principles, the present study primarily highlights the heterogeneity and interpretive variability encountered in retrospective real-world assessment of microcalcification-positive lesions. Prospective multicenter studies applying contemporary BI-RADS descriptors and including both biopsied and non-biopsied lesions are warranted for further validation.

### Clinical implications

From a clinical perspective, our findings should be interpreted as supplementary evidence from a pathology-enriched retrospective cohort rather than as a replacement for established BI-RADS principles. These results may help illustrate the heterogeneity of microcalcification-positive lesions encountered in routine practice, particularly among lesions initially assigned to BI-RADS 4 A in our institutional setting, and may support more cautious interpretation in selected cases.

In addition, these observations may provide hypothesis-generating information for future studies integrating mammographic imaging features into artificial intelligence– or radiomics-assisted diagnostic models. Further prospective multicenter validation will be necessary before such approaches can be translated into broader clinical decision-making.

## Data Availability

The datasets generated and/or analysed during the current study are not publicly available due to patient privacy and ethical restrictions but are available from the corresponding authors on reasonable request.
